# The complete mitochondrial genome of the cigarette beetle, *Lasioderma serricorne* (Coleoptera: Anobiidae)

**DOI:** 10.1080/23802359.2017.1357448

**Published:** 2017-07-25

**Authors:** Wen-Jia Yang, Kang-Kang Xu, Xiao-Ye Zhu, Chun-Xu Chen, Xing-Ying Cai, Yu Cao, Yong-Lu Meng, Hong Yang, Can Li

**Affiliations:** a Key & Special Laboratory of Guizhou Education Department for Pest Control and Resource Utilization, College of Biology and Environmental Engineering, Guiyang University, Guiyang, China;; b Institute of Entomology, College of Tobacco Science, Guizhou University, Guiyang, China

**Keywords:** *Lasioderma serricorne*, cigarette beetle, mitochondrial genome

## Abstract

The cigarette beetle, *Lasioderma serricorne* (Fabricius), is an important pest of stored commodities and distributed widely in the world. Here, we report the complete mitochondrial genome of *L. serricorne* which was 15,958 bp and composed of 13 protein-coding genes (PCGs), two rRNA genes, 22 tRNA genes and a control region. The gene order and orientation of *L. serricorne* were identical to those of other Coleopteran mitogenomes. ATG, ATA, ATT, ATC, TTG were initiation codons and TAA, TAG, T were termination codons. All 22 tRNA genes were predicted with a typical cloverleaf structure except for *trnS_1_* (AGN). Phylogenetic analysis performed using 13 PCGs with 14 other beetles showed that *L. serricorne* is closely related to *Stegobium paniceum*, which agree with the conventional taxonomy.

The cigarette beetle, *Lasioderma serricorne* (Fabricius) (Coleoptera: Anobiidae), is a major pest in tobacco and other stored commodities, which is of important economic value and distributed widely in the world (Li et al. 2009; Imai 2015). The samples of *L. serricorne* were collected from tobacco warehouse in Guizhou province of China (N26°30′, E106°40′), and stored in the insect specimen room of Guiyang University with an accession number GYU-Col-20090002-2.

The complete mitochondrial genome of *L. serricorne* (GenBank accession No. MF417629) is a closed circular molecule of 15,958 bp in length, with the typical gene content as other metazoan mitogenomes, including 13 protein-coding genes (PCGs), two ribosomal RNA genes (*rrnL* and *rrnS*), 22 transfer RNA (tRNA) genes, and a putative control region (Boore 1999). The overall base composition of *L. serricorne* mitogenome was A (38.16%), T (40.44%), G (10.32%), and C (11.08%). The AT-skew and GC-skew of this genome were –0.029 and –0.036, respectively. The gene order and orientation of *L. serricorne* were identical to those observed in other Coleopteran mitogenomes. Twenty-four genes were transcribed on the majority strand (J-strand), whereas the others were oriented on the minority strand (N-strand). The *L. serricorne* mitogenome harbours a total of 31 bp intergenic spacer sequences, which is made up of 8 regions in the range from 1 to 17 bp. The largest intergenic spacer sequence of 17 bp is located between *trnS_2_* and *nad1*. Gene overlaps were found at 15 gene junctions and involved a total of 42 bp, the longest 8 bp overlapping located between *trnW* and *trnC*. The control region was located between *rrnS* and *trnI* gene with a length of 1457 bp, and the A + T content was 80.44%. This region consisting of two tandem repeat sequences, both of them contain three 227 bp repeat units.

With an exception for *trnS_1_* (AGN), all tRNA genes have the conventional cloverleaf secondary structure, which are common in most animal mitogenomes (Wolstenholme 1992). The length of these tRNAs ranged from 60 bp (*trnC*) to 71 bp (*trnK*), A + T content ranged from 70.31% (*trnY*) to 90.48% (*trnE*). The *rrnL* was located between *trnL_1_* and *trnV*, *rrnS* resided between *trnV* and the control region. The lengths of *rrnL* and *rrnS* in the *L. serricorne* mitogenome were 1261 bp and 769 bp, respectively; and the A + T contents were 82.87% and 80.23%, respectively, which were consistent with those reported in other beetles (Liu et al. 2014; Yang et al. 2016; Yuan et al. 2016).

Among 13 PCGs, eight genes share the complete termination codon TAG or TAA, and the remaining PCGs including *cox1*, *cox2*, *cox3*, *nad4*, and *nad5* use a single T as stop codon. The initial codons for 11 PCGs of *L. serricorne* were the canonical putative start codons ATN (ATG for *atp6*, *cox3*, *nad4L*, and *cob*; ATT for *nad2*, *nad5*, and *atp8*; ATA for *nad1*, *nad4*, and *nad6*; ATC for *nad3*). However, *cox1* and *cox2* used AAT and TTG as start codon, respectively. Based on the concatenated amino acid sequences of 13 PCGs, the neighbour-joining method was used to construct the phylogenetic relationship of *L. serricorne* with 14 other representative beetles. The results demonstrated that *L. serricorne* is closely related to *Stegobium paniceum* (Figure 1), which agree with the conventional taxonomy.

**Figure 1.**
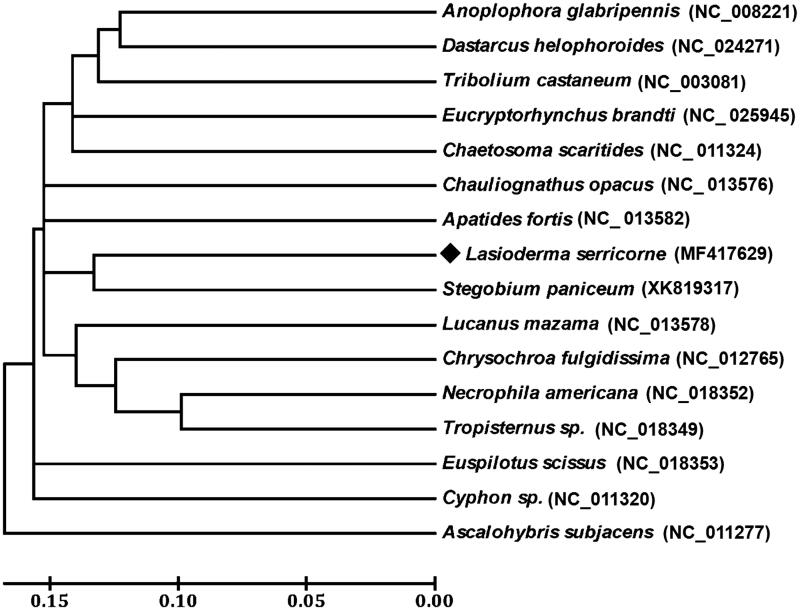
Phylogenetic tree showing the relationship between *L. serricorne* and 14 other beetles based on neighbour-joining method. *Ascalohybris subjacens* was used as an outgroup. GenBank accession numbers of each species were listed in the tree.
